# Identification of a Novel *GOLGB1-RET* Fusion in Papillary Thyroid Cancer With a Durable Response to Selpercatinib

**DOI:** 10.1200/PO-26-00237

**Published:** 2026-07-01

**Authors:** Shogen Boku, Takao Fujisawa, Shigenori Kadowaki, Hironaga Satake, Hisateru Yasui, Koushiro Ohtsubo, Yasushi Shimizu, Tomoyuki Otsuka, Bunya Kuze, Riu Yamashita, Taro Shibuki, Yoshiaki Nakamura, Hideaki Bando, Takayuki Yoshino, Milan Radovich, Susumu Okano

**Affiliations:** ^1^ Department of Clinical Oncology, Kansai Medical University Hospital, Hirakata City, Osaka, Japan; ^2^ Department of the Promotion of Drug and Diagnostic Development, National Cancer Center Hospital East, Kashiwa City, Chiba, Japan; ^3^ Department of Head and Neck Medical Oncology, National Cancer Center Hospital East, Kashiwa City, Chiba, Japan; ^4^ Department of Clinical Oncology, Aichi Cancer Center Hospital, Chikusa-ku, Nagoya City, Aichi, Japan; ^5^ Department of Medical Oncology, Kochi Medical School, Kohasu, Oko-cho, Nankoku City, Kochi, Japan; ^6^ Department of Medical Oncology, Kobe City Medical Center General Hospital, Chuo-ku, Kobe City, Hyogo, Japan; ^7^ Department of Medical Oncology, Kanazawa University Hospital, Kanazawa City, Ishikawa, Japan; ^8^ Department of Medical Oncology, Hokkaido University Hospital, Kita-ku, Sapporo, Hokkaido, Japan; ^9^ Department of Medical Oncology, Osaka International Cancer Institute, Chuo-ku, Osaka City, Osaka, Japan; ^10^ Department of Head and Neck Surgery, Central Japan International Medical Center, Minokamo, Gifu, Japan; ^11^ Division of Translational Informatics, Exploratory Oncology Research and Clinical Trial Center, National Cancer Center, Kashiwa City, Chiba, Japan; ^12^ Department of Hepatobiliary and Pancreatic Oncology, National Cancer Center Hospital East, Kashiwa City, Chiba, Japan; ^13^ Department of Gastroenterology and Gastrointestinal Oncology, National Cancer Center Hospital East, Kashiwa City, Chiba, Japan; ^14^ Caris Life Sciences, Phoenix, AZ

## Introduction

Papillary thyroid carcinoma (PTC) is the most common endocrine malignancy worldwide. Most patients do well with surgery and radioactive iodine (RAI), but a subset develops RAI-refractory disease that requires systemic therapy.^1,2^ Multikinase inhibitors (MKIs), such as lenvatinib, offer benefits that are limited by off-target toxicities and resistance.^3^

*RET* fusion is a recurrent driver of PTC. Large cohorts estimate a prevalence of 7%-9% in adults, with variation by assay and population.^4-6^ The most common *RET* fusion partners in PTC are *CCDC6* and *NCOA4*, historically designated *RET*/*PTC1* and *RET*/*PTC3*, respectively. Recent sequencing studies have expanded this spectrum by identifying additional rare *RET* fusion partners, including *PRKAR1A*, *SQSTM1*, *TPR*, *GOLGA5*, *ACBD5*, *RUFY2*, and others.^7^ However, *GOLGB1* has not, to our knowledge, been previously reported as a *RET* fusion partner in PTC or other thyroid malignancies. This distinction is clinically relevant because rare or novel fusion partners may be missed by assays designed around predefined breakpoints or partner genes.

Selective RET inhibition has changed the management of *RET* fusion–positive thyroid cancer. Selpercatinib produced high response rates and durable control in *RET*-altered thyroid cancers in LIBRETTO-001, and now has indications that include *RET* fusion–positive thyroid cancer and a tissue-agnostic label for *RET* fusion–positive solid tumors.^8^ Assay selection is a critical determinant of fusion detection sensitivity. Amplicon-based next-generation sequencing (NGS) often targets predefined breakpoints and partners, and can miss rare or novel events. Hybrid-capture DNA profiling and RNA sequencing identify rearrangements without prior knowledge and are preferred when initial testing is negative, despite clinical suspicion.^9-11^

Here, we report a patient with RAI-refractory PTC in whom *GOLGB1-RET* was discovered only after comprehensive profiling, leading to a durable response to selpercatinib.

## Case Presentation

A 41-year-old woman underwent subtotal thyroidectomy with D1 lymph node dissection for PTC (pT3N0M1) with lung metastases. She subsequently received three sessions of RAI therapy, with administered activities of 100, 130, and 130 mCi, corresponding to a cumulative activity of 13.3 GBq (360 mCi). Eighteen months later, radiographic progression after repeated RAI therapy, together with intense fluorodeoxyglucose positron emission tomography uptake, supported the diagnosis of RAI-refractory disease. Targeted profiling of the primary tumor formalin-fixed paraffin-embedded (FFPE) block with Oncomine Dx Target Test Multi-CDx (amplicon-based multiplex NGS) detected no actionable alterations, including *RET* fusions. Owing to local recurrence with tracheal invasion, lenvatinib 14 mg/day was initiated, achieving early tumor shrinkage. The treatment was complicated by grade 2 hepatic injury, requiring interruption and dose reduction to 4 mg/day.

**FIG 1.**
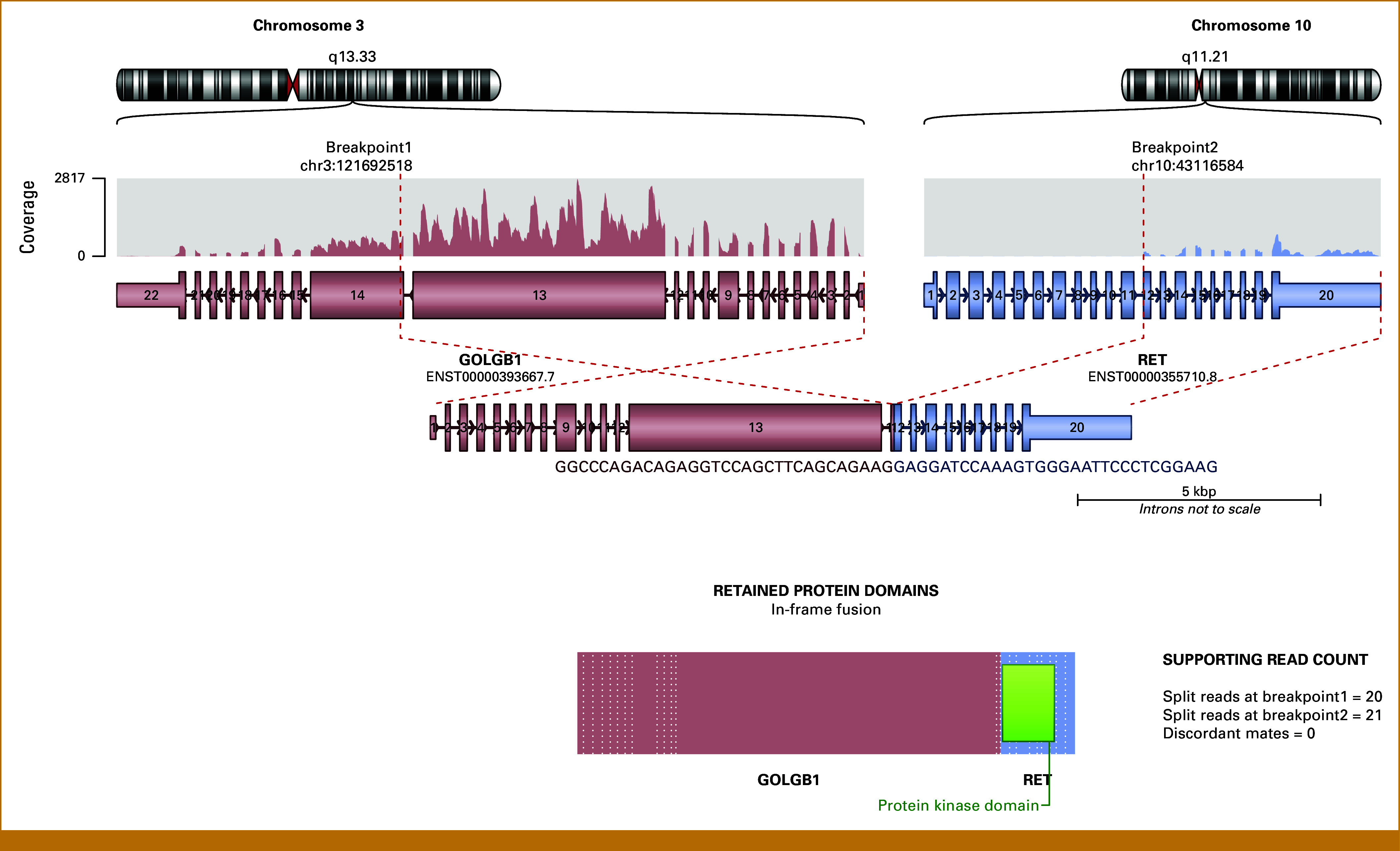
Identification and structure of the *GOLGB1-RET* fusion. Schematic representation of the in-frame *GOLGB1-RET* fusion detected by whole-transcriptome sequencing. The fusion joins *GOLGB1* exon 13 to *RET* exon 12 and retains the RET protein kinase domain. RNA-seq coverage tracks indicate read depth, with a maximum depth of 2,817 reads at chr3:121692518. The junction was supported by 20 and 21 split reads at breakpoints 1 and 2, respectively, with no discordant mate reads, supporting an expressed in-frame fusion transcript.

After 7 months, imaging confirmed disease progression. To explore further therapeutic options within a research framework, the same FFPE block was reanalyzed using the SCRUM-Japan MONSTAR-SCREEN-2 platform.^12^ Whole-transcriptome sequencing (WTS) identified an in-frame, intragenic *GOLGB1-RET* fusion transcript joining *GOLGB1* exon 13 to *RET* exon 12 (Fig 1). Prompted by this research discovery, subsequent FoundationOne CDx (hybrid-capture DNA profiling) detected *RET* rearrangements, enabling access to selpercatinib under insurance coverage. FoundationOne CDx also detected a TERT promoter -124C>T alteration. Microsatellite status was stable, and tumor mutational burden was 5 mutations/Mb. No additional established actionable alteration was reported. The sample qualified for suboptimal assessment of copy-number alterations; therefore, sensitivity for detecting gene amplifications and losses may have been reduced.

**FIG 2.**
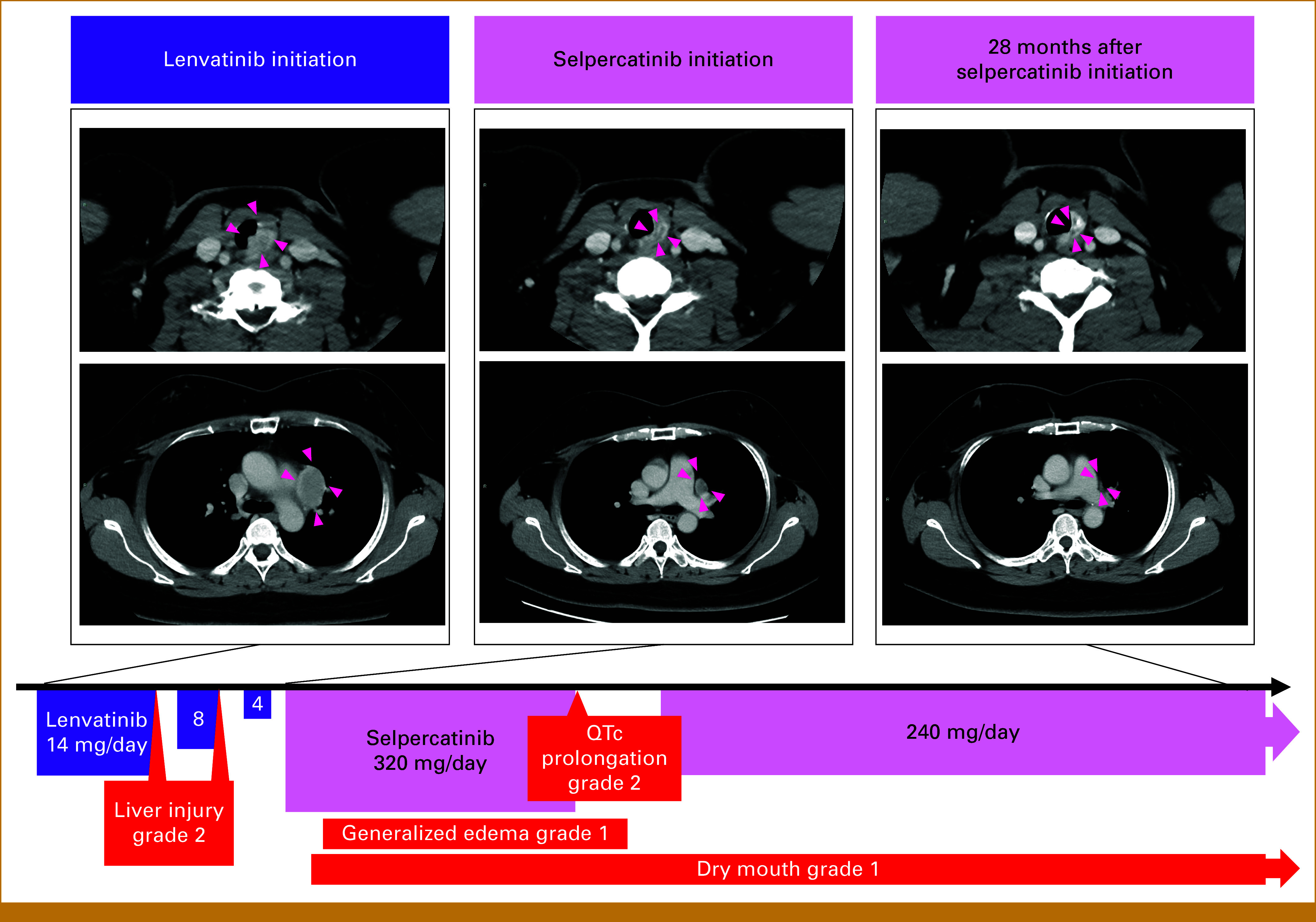
Radiologic response to selpercatinib. Representative computed tomography images show shrinkage of the neck and mediastinal lesions after selpercatinib initiation. The timeline summarizes systemic therapies, genomic assays, and adverse events.

Selpercatinib was administered as second-line therapy. During treatment, grade 2 QTc prolongation required dose reduction; notably, hepatotoxicity did not recur. A retrospective radiologic assessment according to RECIST version 1.1 confirmed partial response: the sum of target lesion diameters decreased from 40 mm at baseline to 11 mm at best response, corresponding to a 72.5% reduction. Serial computed tomography at 28 months showed a sustained partial response in the neck and mediastinal disease (Fig 2). The patient remains on selpercatinib, with ongoing disease control.

## Discussion

The present case provides clinical evidence that *GOLGB1-RET* is a therapeutically actionable fusion in PTC. The radiographic tumor shrinkage observed with selpercatinib after intolerance to lenvatinib supports a causal role of fusion in driving disease biology.

*GOLGB1* (giantin) is a large coiled-coil Golgi matrix protein. Coiled-coil–mediated dimerization is a recurring mechanism in kinase fusion oncogenes that promotes ligand-independent activation. Several *GOLGB1* kinase fusions have been reported—*GOLGB1-PDGFRB*, and *GOLGB1-FLT3*—each using the 5′ partner's dimerization motifs and promoter activity to activate the 3′ kinase domain.^13,14^ In addition, *GOLGB1-BRAF* has been reported in low-grade nasopharyngeal adenocarcinoma, further supporting the recurrent involvement of *GOLGB1* as a 5′ partner in oncogenic kinase fusions.^15^ By incorporating the dimerization motifs of the 5′ partner, the in-frame *GOLGB1-RET* retaining the RET tyrosine kinase domain is expected to signal constitutively through oncogenic pathways.

The initial negative result from the amplicon-based panel highlighted a critical diagnostic pitfall. Amplicon assays typically query predefined junctions; thus, novel partners or atypical intronic breakpoints may be missed. In contrast, partner-agnostic hybrid-capture DNA assays and RNA sequencing (WTS) can identify rearrangements independently of the 5′ partner, making them essential reflex tests when clinical suspicion remains high despite initial negative results.^9-11^

Furthermore, selective RET inhibition offers a superior therapeutic index for this patient.^8^ Although lenvatinib was constrained by grade 2 hepatotoxicity, selpercatinib was well tolerated without recurrence of liver injury. This underscores the clinical benefit of transitioning from broad MKIs to highly selective targeted therapies, based on comprehensive genomic profiling.

In conclusion, *GOLGB1-RET* is an actionable RET fusion in PTC. Comprehensive genomic testing, preferably hybrid-capture DNA with RNA sequencing, prevents missed therapeutic opportunities and enables the effective use of selpercatinib.

### Ethics Approval and Consent to Participate

The patient provided written informed consent for participation in the SCRUM-Japan MONSTAR-SCREEN-2 project (UMIN000043899) and for publication, in accordance with the Declaration of Helsinki. Separate institutional review board approval for this case report was not required according to institutional policy.

### Consent for Publication

Written informed consent for publication of clinical details and images was obtained.

## Data Availability

Deidentified data supporting the findings of this case report are available from the corresponding author upon reasonable request and subject to applicable ethical and institutional restrictions.
